# Assessing the potential for improved predictive capacity of antimicrobial resistance in outpatient *Staphylococcus aureus* isolates using seasonal and spatial antibiograms

**DOI:** 10.1186/s13756-024-01388-3

**Published:** 2024-03-22

**Authors:** Margaret Carrel, Qianyi Shi, Gosia S. Clore, Shinya Hasegawa, Matthew Smith, Eli N. Perencevich, Michihiko Goto

**Affiliations:** 1 Department of Geographical & Sustainability Sciences, University of Iowa, Iowa City, IA USA; 2 Department of Internal Medicine, University of Iowa, Iowa City, IA USA; 3 Center for Access & Delivery Research and Evaluation (CADRE), Iowa City Veterans Affairs Health Care System, Iowa City, IA USA; 4 Iowa City, USA

**Keywords:** Antibiogram, Staphylococcus aureus, Seasonal, Spatial, Susceptibility

## Abstract

**Background:**

While the use of cumulative susceptibility reports, antibiograms, is recommended for improved empiric therapy and antibiotic stewardship, the predictive ability of antibiograms has not been well-studied. While enhanced antibiograms have been shown to better capture variation in susceptibility profiles by characteristics such as infection site or patient age, the potential for seasonal or spatial variation in susceptibility has not been assessed as important in predicting likelihood of susceptibility.

**Methods:**

Utilizing *Staphylococcus aureus* isolates obtained in outpatient settings from a nationwide provider of care, the Veterans Health Administration, and a local provider of care, the University of Iowa Hospitals and Clinics, standard, seasonal and spatial antibiograms were created for five commonly used antibiotic classes: cephalosporins, clindamycin, macrolides, tetracycline, trimethoprim/sulfamethoxazole.

**Results:**

A total of 338,681 *S. aureus* isolates obtained in VHA outpatient settings from 2010 to 2019 and 6,817 isolates obtained in UIHC outpatient settings from 2014 to 2019 were used to generate and test antibiograms. Logistic regression modeling determined the capacity of these antibiograms to predict isolate resistance to each antibiotic class. All models had low predictive capacity, with areas under the curve of < 0.7.

**Conclusions:**

Standard antibiograms are poor in predicting *S. aureus* susceptibility to antibiotics often chosen by clinicians, and seasonal and spatial antibiograms do not provide an improved tool in anticipating non-susceptibility. These findings suggest that further refinements to antibiograms may be necessary to improve their utility in informing choice of effective antibiotic therapy.

**Supplementary information:**

The online version contains supplementary material available at 10.1186/s13756-024-01388-3.

## Background

As antimicrobial resistance (AMR) increases in all healthcare settings, including outpatient clinics, it is increasingly important to provide accurate information about the risk of AMR to prescribing clinicians. Cumulative susceptibility testing results, or antibiograms, is a commonly used epidemiologic method to track trends in drug resistance prevalence, guide antimicrobial empiric therapy and improve antibiotic stewardship [1–3]. Known limitations associated with antibiograms include wide variability in how antibiograms are prepared and varying confidence and ability by clinicians to interpret antibiogram information [4–12]. What is less well known, however, is whether antibiograms accurately predict resistance when empiric therapeutic decisions are made; research examining the diagnostic accuracy of antibiograms for Enterobacteriaceae isolates in a nationwide cohort in the United States (US) suggests their guidance may be poor [13].

In the US, the recommendations for antibiograms set forth by the Clinical & Laboratory Standards Institute (CLSI) include antibiogram construction at least annually and reporting results only for ≥ 30 isolates per species aggregated by facility [14, 15]. Enhanced antibiograms, where results are stratified by patient characteristics (e.g., age), culture site (e.g., urine or blood), and location (e.g., inpatient or outpatient), have been proposed to improve accuracy in guiding empiric therapy decisions, though these stratifications can result in samples sizes below the ≥ 30 isolate minimum or prove challenging for some facilities to generate [16–23].

The prevalence of bacterial infections and rates of antibiotic resistance vary seasonally and geographically in the US and globally [24–29]. Standard antibiograms do not consider the potential impacts of seasonality on AMR patterns or the varying geographic locations from which patients are drawn. Many outpatient clinics are distant from affiliated medical centers where they aggregate data, masking potential variation in environmental exposures or local circulation of resistance types, or are not affiliated with any hospital at all, meaning that AMR patterns in outpatient settings are not summarized for clinicians. Creating geography- or season-specific antibiograms could potentially improve the quality of information available to clinicians and thus improve empiric therapy decisions and antibiotic stewardship.

Utilizing curated pre-pandemic microbiology test results from the only integrated nationwide healthcare system in the US, the Veterans Health Administration (VHA), we compare the predictive accuracy of seasonal and spatial antibiograms to standard antibiograms for *Staphylococcus aureus* infections observed in outpatient settings. We also analyzed data from a regional provider of care, the University of Iowa Hospitals & Clinics (UIHC), to test the generalizability of the approach beyond VHA data.

## Methods

The VHA’s Corporate Data Warehouse (CDW) was queried for all *S. aureus* clinical specimens from outpatient settings from 1/1/2010-12/31/2019. Isolates were included from all sources and sites, not only for invasive infections, given the outpatient nature of the cohort. Specimens were then excluded if they were missing geographic information (e.g., address, longitude/latitude, or county of residence) if they were obtained from a patient residing outside of the conterminous 48 states or Washington, DC (CONUS) if they were from a patient < 18 years of age or if they were obtained > 48 h after admission to institutionalized settings (e.g., nursing home, acute care) and < 72 h after discharge (see Additional Fig. 1). Clinical specimen records were then linked to microbiological results; if these susceptibility results were missing, the record was excluded. Microbiology reports were used to assess susceptibility or resistance; intermediate resistance was classified as resistant. Similar exclusion criteria were applied to *S. aureus* clinical specimen records extracted from the Epic electronic health records system of the UIHC from 1/1/2014-12/31/2019 (see Additional Fig. 2). Data from 2010 to 2014 was not available from UIHC due to changes in recordkeeping that occurred. UIHC *S. aureus* specimens from pediatric patients were retained to more accurately reflect patient distributions in US healthcare facilities than is observed in the VHA cohort and since standard antibiograms do not stratify by patient age.

For each of the datasets, three sets of antibiograms were generated for five antibiotic classes commonly used in outpatient settings: 1st -4th generation cephalosporins, clindamycin, macrolides, tetracycline and trimethoprim/sulfamethoxazole (TMP/SMX). Cephalosporin resistance is a surrogate for methicillin resistance (MRSA) as most clinical microbiology laboratories within the VHA system do not perform phenotypic susceptibility testing for methicillin or oxacillin anymore, rather they use susceptibility to 1st generation cephalosporin or cefoxitin to determine MRSA vs. MSSA. For standard antibiograms, the proportion of *S. aureus* isolates, one per patient per year, susceptible to each of the five classes was calculated in each year for each of the 138 outpatient facilities of the VHA and for the single UIHC facility. For seasonal antibiograms, susceptibility results were aggregated into four three-month seasons (December, January, February; March, April, May; June, July, August; September, October, November) across the ten years of the study, resulting in potentially forty antibiograms per facility for the VHA data and twenty-four antibiograms for the single UIHC facility. Only the first *S. aureus* isolate per patient in each season/year was used. Spatial antibiograms were generated based upon county residence of the patient at an annual scale using the first patient record per year in each county. The potential maximum number of antibiograms is thus ten for each of the > 3000 CONUS counties where *S. aureus* isolates were obtained from US veterans and five for each of the 100 counties where *S. aureus* isolates were obtained from UIHC patients.

To be consistent with the CLSI recommendations to include only routinely tested antimicrobial agents, antibiograms were generated only for antibiotic classes where susceptibility was reported for > 90% of isolates per year [14]. This results in varying numbers of standard, seasonal and spatial antibiograms. To further follow CLSI recommendations, we also generated county-level, facility/year and season/year antibiograms only when 30 or more eligible isolates were available for analysis and assessed their predictive capacity separately from the entire dataset [14].

The predictive power of seasonal or spatial antibiograms versus standard antibiograms was assessed via logistic regression modeling. The modeled outcome was whether an isolate was resistant to a certain antibiotic (e.g., clindamycin), while the independent variable was the prevalence of susceptible isolates reported in the antibiogram at predefined thresholds. Thresholds of susceptibility that were examined were < 80%, < 85%, < 90%, < 95% and < 98%, based upon surveys of infectious disease specialists that indicated altered decision-making in prescribing choices at susceptibility thresholds of 85–95% [30]. For example, the likelihood of clindamycin non-susceptibility in a *S. aureus* isolate collected in October 2018 from a patient residing in Johnson County, Iowa seen at UIHC would be predicted by the prevalence of clindamycin susceptibility in *S. aureus* isolates observed at UIHC in 2017, in July/August/September of 2018 and in Johnson County in 2017 at thresholds of AMR from 2 to 20%. Predictive capacity is summarized by the area under the curve (AUC); AUC > 0.9 indicate high predictive capacity, 0.8–0.9 are good, 0.7–0.8 are moderate, and AUC < 0.7 are poor. Sensitivity and specificity were calculated at each threshold for each model.

In addition to assessing predictive capacity, we also used VHA data to assess how many patients have county-level antibiograms available. We calculated annual proportions of patients who had county-level antibiograms available for their county of residence from the previous time frame (e.g., previous calendar year) among all patients who utilized VHA care during the respective years. This provides insight into the generalizability of this approach in counties with smaller populations.

All statistical analyses were performed with R version 4.3.1 (R Foundation for Statistical Computing, Vienna, Austria). Ethical approval for the study was obtained from the Iowa City Veterans Affairs Health Care System and University of Iowa Institutional Review Boards.

## Results

A total of 338,681 *S. aureus* isolates obtained in VHA outpatient settings from 2010 to 2019 and 6,817 isolates obtained in UIHC outpatient settings from 2014 to 2019 were used to generate and test antibiograms. Patient demographics from VHA differed from that of UIHC: the VHA dataset was majority male while the UIHC data was more evenly split according to gender, and the UIHC dataset had approximately one-fifth of samples from pediatric patients while these were excluded from the VHA dataset (*n* = 2) (Table 1).

**Table 1:** Demographic characteristics of patients with positive *S. aureus* outpatient cultures in the VHA and UIHC.

	VHA		UIHC	
Variable	N	%	N	%
Total	338,681		6817	
Gender				
Female	18,084	5.3%	3151	46.2%
Male	320,597	94.7%	3666	53.8%
Age				
< 18	0	0.0%	1564	22.9%
18–35	16,950	5.0%	1295	19.0%
35–50	33,701	10.0%	1054	15.5%
50–65	122,536	36.2%	1540	22.6%
65–80	120,678	35.6%	1035	15.2%
80+	44,816	13.2%	329	4.8%

VHA: Veterans Health Administration; UIHC: University of Iowa Hospitals & Clinics

Though the number varied by antibiotic class and temporally, VHA isolates were drawn from a range of 84–136 of the 138 outpatient VHA facilities and from 2337 to 2836 of the 3,038 counties in the CONUS with outpatient veteran visits (Table 2). UIHC data came from a single facility and 98–100 counties, predominantly in Iowa and Western Illinois. When the data was subset to consider only facilities or counties with ≥ 30 isolates, the majority of counties and isolates in the VHA data were excluded, though this varied by antibiotic class. All UIHC *S. aureus* samples were tested against all five antibiotic classes but only ten counties met the ≥ 30 isolate minimum.

**Table 2:** Resistance rates of *S. aureus* to antimicrobial classes in the VHA and UIHC and samples sizes for facilities and counties included in antibiogram generation for the overall datasets and then the subset that met the ≥ 30 isolate CLSI recommendation

	VHA					UIHC				
Antibiotic Group	Overall Resistance	Facilities	Facilities ≥ 30	Counties	Counties ≥ 30	Overall Resistance	Facilities	Facilities ≥ 30	Counties	Counties ≥ 30
Cephalosporins	44.31%	136	130	2836	328	33.15%	1	1	100	10
Clindamycin	23.82%	85	79	2505	201	32.51%	1	1	98	10
Macrolides	59.11%	84	78	2337	194	54.60%	1	1	98	10
Tetracyclines	6.11%	128	123	2686	299	7.45%	1	1	100	10
TMP/SMX	3.09%	130	125	2627	309	3.54%	1	1	100	10

VHA: Veterans Health Administration; UIHC: University of Iowa Hospitals & Clinics

Antibiotic resistance rates for some antibiotic classes differed between the VHA and UIHC datasets. Cephalosporin resistance was over 10% higher in VHA *S. aureus* samples than in UIHC, and macrolides resistance was 5% higher. Clindamycin resistance in UIHC samples was 8% higher than in VHA *S. aureus*. Resistance rates for tetracyclines and TMP/SMX were comparable.

All antibiograms performed poorly in predicting resistance, across all five antibiotic classes, with AUC below 0.7 (Table 3). Including only facilities, counties or seasons with ≥ 30 isolates did not significantly improve predictive ability. Standard antibiograms had the greatest predictive capacity for VHA datasets while spatial antibiograms had the greatest predictive capacity for UIHC datasets. Seasonal, facility level antibiograms performed the worst in all trials. Higher, though still very poor, predictive ability was observed for antibiotic classes with the highest levels of susceptibility. This is reflected in reasonable sensitivity and specificity levels at high (> 90%) thresholds only for tetracyclines and TMP/SMX and only in the VHA models with large enough sample sizes (Additional Table 1).

**Table 3:** Performance metrics for standard, seasonal and spatial antibiograms generated for five antimicrobial classes for overall datasets and then those facilities/counties that met the ≥ 30 isolate CLSI recommendation

	Antibiotic	Standard	Seasonal	Spatial
*VHA*	Cephalosporins	0.581	0.577	0.563
	Clindamycin	0.554	0.540	0.543
	Macrolides	0.543	0.541	0.541
	Tetracyclines	0.571	0.553	0.551
	TMP/SMX	0.653	0.641	0.630
*VHA* ≥ *30*	Cephalosporins	0.583	0.573	0.571
	Clindamycin	0.550	0.543	0.539
	Macrolides	0.551	0.543	0.543
	Tetracyclines	0.564	0.544	0.555
	TMP/SMX	0.657	0.641	0.646
*UIHC*	Cephalosporins	0.513	0.495	0.552
	Clindamycin	0.504	0.507	0.529
	Macrolides	0.514	0.530	0.539
	Tetracyclines	0.550	0.515	0.564
	TMP/SMX	0.578	0.533	0.616
*UIHC* ≥ *30*	Cephalosporins	0.513	0.496	0.534
	Clindamycin	0.504	0.507	0.558
	Macrolides	0.513	0.530	0.571
	Tetracyclines	0.550	0.515	0.559
	TMP/SMX	0.578	0.533	0.651

VHA: Veterans Health Administration; UIHC: University of Iowa Hospitals & Clinics

The proportion of patients who would have annual county-level antibiograms from the previous calendar year to use in predicting susceptibility among the entire patient population for each calendar year ranged from 44.7 to 49.0% (mean: 46.8%).

## Discussion

In the analysis of the potential for seasonal and spatial antibiograms to improve the capacity to predict non-susceptibility of *S. aureus* to five antibiotic classes, models built using > 330,000 isolates from patients across the US seen in outpatient VHA facilities and using > 6000 isolates from patients seen in outpatient UIHC facilities performed poorly, on par with flipping a coin. Antibiograms created according to current CLSI standards for each facility (ignoring geographic information) were similarly inaccurate in predicting *S. aureus* susceptibility. The poor performance of standard antibiograms using VHA data has previously been observed for Enterobacteriaceae [13]. While UIHC’s patient population is less spatially representative than VHA, it is more balanced in terms of gender and age, but antibiograms generated using this more diverse patient dataset still performed poorly.

When the ≥ 30 minimum was implemented, the number of counties, and by extension isolates, included in the analysis declined greatly for both the VHA and UIHC dataset and the coverage of the total veteran population residing in included counties fell from 95 to 47%. Thus, even for the largest provider of healthcare in the US, generating spatial antibiograms while following the CLSI recommendation only to use data with 30 or more isolates is difficult. The creation of community-level antibiograms, ranging from facilities in a single county to multi-county, has been suggested as a tool for facilities that may otherwise fail to meet the ≥ 30 isolate minimum [31, 32]. This, however, poses a challenge for greater granularity in considering the social and environmental characteristics of places where patients reside and how this can impact the susceptibility profiles of their infections. Determining best practices to meet the CLSI recommended isolate numbers is necessary; for example, using spatial smoothing or aggregating facilities/counties based upon shared sociodemographic or environmental characteristics rather than adjacency might improve the predictive capabilities of antibiograms.

The strength of this study includes the use of a large cohort from the only integrated healthcare system with a presence in all conterminous states in the US, the VHA, with more than 300,00 isolates. The standardized data extraction process and aggregation methods, rather than reliance on locally produced antibiograms, can avoid the problem from the practice variation in antibiogram preparation. A previous study indicated that only a small fraction of facilities are completely adhering to the CLSI guidelines [10]. Studies that assessed antibiogram generation and usage in other nations or regions are sparse, but some suggested this situation might be similar in European nations where guidelines published by the European Committee on Antimicrobial Susceptibility Testing (EUCAST) are used [33].

There are several limitations to this study. First, the VHA provides care to more elderly, male-dominant populations compared to the nationwide demographics of the US. Previous studies suggested that males have a higher risk of methicillin-resistant *S. aureus* (MRSA) carriage and invasive infections, but data for gender differences in resistance prevalence for other antimicrobial classes are sparse [34]. This may potentially limit the generalizability to populations outside the VHA. However, while the proportion of females has increased gradually over the years, there has been no drastic and sudden change during the study period [35]. Additionally, the UIHC cohort more closely resembles the age and gender profile of the US and models using this dataset performed similarly to the VHA data. Second, we focused on assessing standardized antibiograms based on the CLSI guidelines and did not compare them with antibiograms generated by other standards, such as the EUCAST guidelines.

## Conclusions

Although seasonal and spatial antibiograms performed as or more poorly than standard antibiograms, spatiotemporal variation in infection prevalence and antibiotic resistance coupled with observed variation in antibiograms that incorporate patient-level and other information suggest that there is utility in learning how to build better antibiograms [18, 20, 22, 25–27, 29]. Future work will merge information on patients with known characteristics of their infections and data from the communities they were drawn to develop models to better predict antibiotic susceptibility. This, coupled with ongoing work in revising how antibiograms are visualized and improving their useability, means that antibiograms may yet become the tools to guide empiric therapy and improve antibiotic stewardship for which they are currently recommended [8, 36–38].

### Electronic supplementary material

Below is the link to the electronic supplementary material.

**Supplementary additional table. 1.** Sensitivity (sn) and specificity (sp) of standard, seasonal and spatial antibiograms predicting non-susceptibility of S. aureus isolates at predefined thresholds.

**Supplementary additional fig. 1.** The creation process of standardized, spatial and seasonal antibiograms for the VHA dataset.

**Supplementary additional fig. 2.** The creation process of standardized, spatial and seasonal antibiograms for the UIHC dataset.

## Data Availability

The datasets generated in this study are not publicly available due to their sensitive nature and ownership by the VHA and UIHC. Access to the datasets may be granted by VHA or UIHC.

## Abbreviations

AMR: Antimicrobial resistance.
CDW: Corporate Data Warehouse.
CLSI: Clinical & Laboratory Standards Institute.
CONUS: Conterminous US (48 states and Washington, DC).
EUCAST: European Committee on Antimicrobial Susceptibility Testing.
MRSA: Methicillin-resistant *S. aureus*.
TMP/SMX: Trimethoprim/Sulfamethoxazole.
UIHC: University of Iowa Hospitals & Clinics.
US: United States.
VHA: Veterans Health Administration.
