# Supplementary figures and images for: Assessing the potential for improved predictive capacity of antimicrobial resistance in outpatient Staphylococcus aureus isolates using seasonal and spatial antibiograms
Source: Antimicrob Resist Infect Control. 2024 Mar 22;13:34. doi: 10.1186/s13756-024-01388-3 (PMC10960453; doi:10.1186/s13756-024-01388-3)

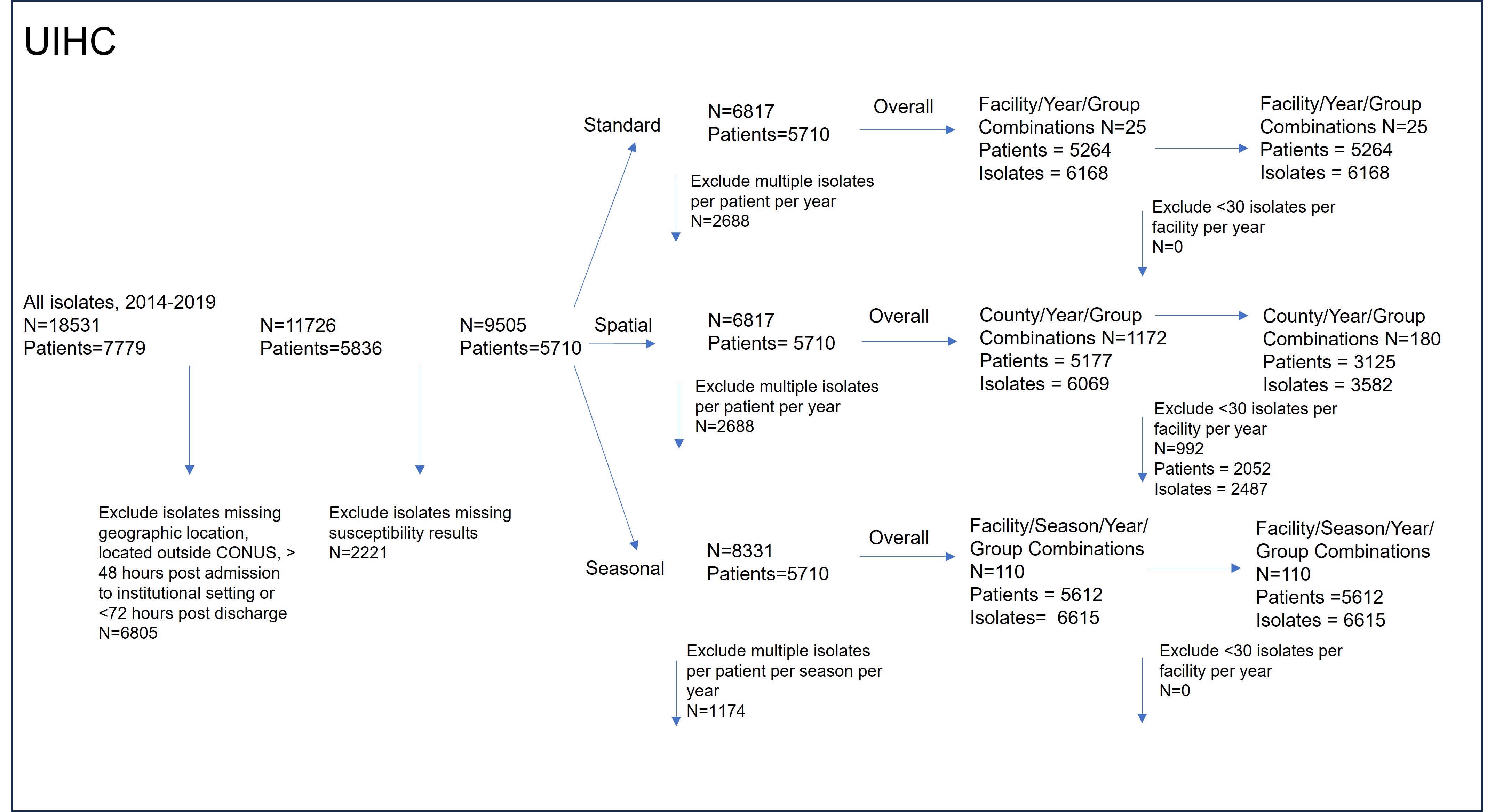

Supplement: Supplementary file 2 — Supplementary additional fig. 1. The creation process of standardized, spatial and seasonal antibiograms for the VHA dataset. [file 13756_2024_1388_MOESM2_ESM.jpg]

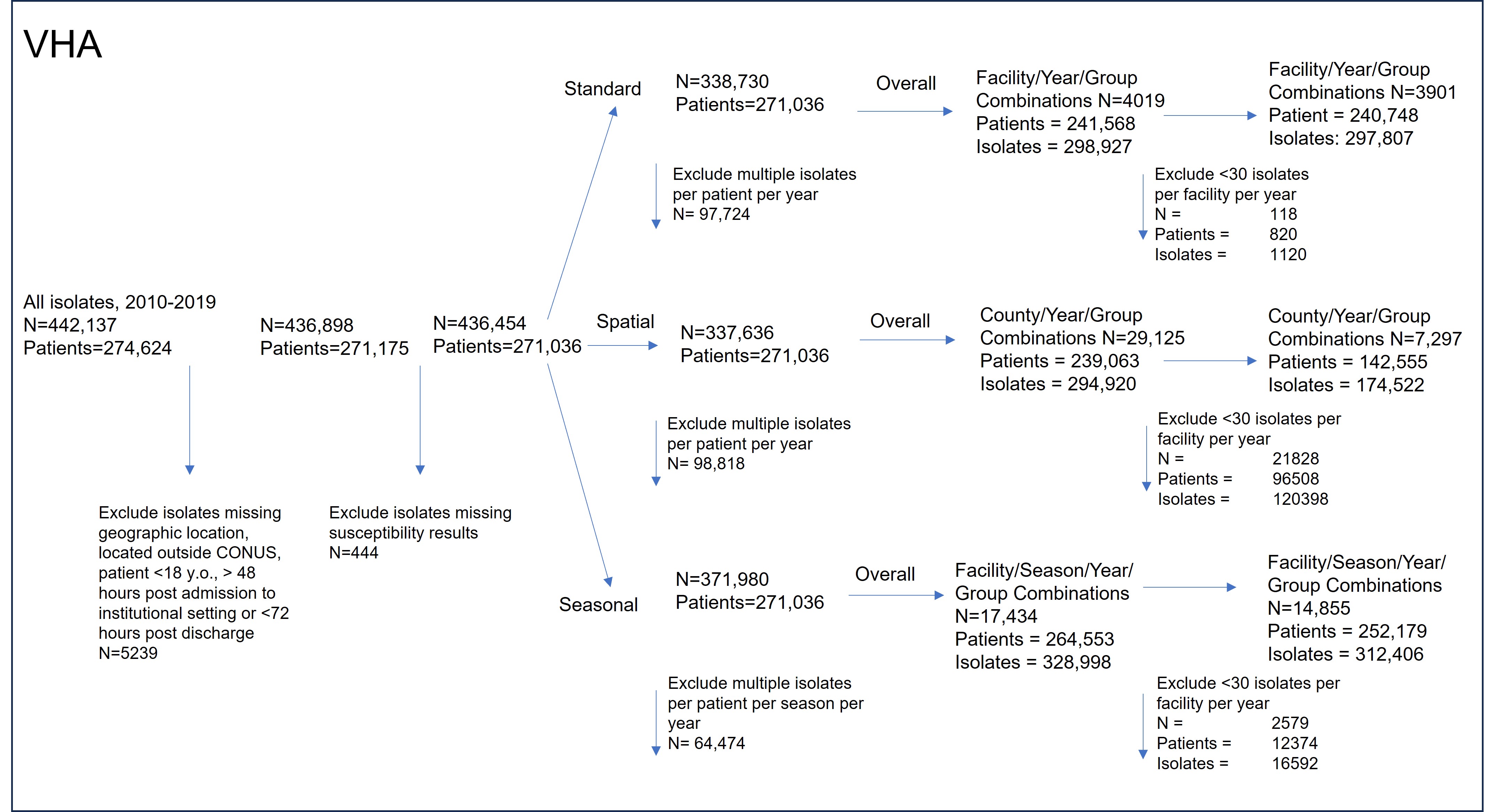

Supplement: Supplementary file 3 — Supplementary additional fig. 2. The creation process of standardized, spatial and seasonal antibiograms for the UIHC dataset. [file 13756_2024_1388_MOESM3_ESM.jpg]
